# Effect of androgen abuse on sleep apnea in men: a prospective cohort study

**DOI:** 10.1007/s40618-026-02943-y

**Published:** 2026-07-20

**Authors:** Tijs Johannes Verdegaal, Wout Peeters, Peter Bond, Herwin Machiels, Willem de Ronde, Diederik Laurens Smit

**Affiliations:** 1 Department of Internal Medicine, Elisabeth-TweeSteden Hospital, Hilvarenbeekseweg 60, 5022 GC Tilburg, The Netherlands; 2 Department of Performance and Image-Enhancing Drugs Research, Android Health Clinic, Leidse Rijn 31, 3454 PZ Utrecht, The Netherlands; 3 Department of Internal Medicine, Spaarne Gasthuis, Boerhaavelaan 22, 2035 RC Haarlem, The Netherlands; 4 Department of Endocrinology and Metabolism, Amsterdam UMC, Meibergdreef 9, 1105 AZ Amsterdam, The Netherlands; 5 Faculty of Medicine, Utrecht University, Heidelberglaan 100, 3584 CX Utrecht, The Netherlands; 6 Department of Pulmonary Medicine, Elisabeth-TweeSteden Hospital, Hilvarenbeekseweg 60, 5022 GC Tilburg, The Netherlands; 7 Department of Internal Medicine, Zaandam Medical Centre, Koningin Julianaplein 58, 1502 DV Zaandam, The Netherlands

**Keywords:** Anabolic steroids, Androgen abuse, Performance and image-enhancing drugs, Sleep apnea

## Abstract

**Purpose:**

Androgen abuse has been suggested to adversely affect sleep-disordered breathing, but prospective real-world data in androgen abusers are lacking. The aim of this study was to evaluate the effects of androgen abuse on sleep-disordered breathing.

**Methods:**

Adult men planning a self-initiated androgen cycle were evaluated before, at approximately week 8 of their cycle, and approximately three months after cycle cessation. Sleep was assessed using ambulatory WatchPAT monitoring. The primary outcome was change in the peripheral arterial tone–derived apnea–hypopnea index (pAHI). Secondary outcomes included peripheral arterial tone-derived respiratory distress index (pRDI) and oxygen desaturation index (pODI), sleep architecture and subjective sleep questionnaires as measured through the Epworth Sleepiness Scale (ESS) and Insomnia Severity Index (ISI). Longitudinal changes were analyzed using linear mixed-effects models.

**Results:**

Twenty-three participants were included. Median cycle duration was 12 weeks with a median weekly dose of 678 mg. During the androgen cycle, pAHI did not increase significantly, whereas pRDI did increase (mean increase of 3.0 events/hour). At post-cycle follow-up, pAHI, pRDI, and pODI were significantly increased compared with baseline (mean increases of 2.7, 4.4, and 3.0 events/hour, respectively). Sleep architecture was largely preserved, although a modest reduction in deep sleep and an increase in heart rate was observed during androgen abuse. No significant changes were observed in total ESS or ISI scores across time points.

**Discussion:**

These findings suggest that androgen abuse may exert subtle effects on sleep-disordered breathing parameters but are unlikely to translate into overt or clinically meaningful sleep apnea in most androgen abusers.

**Clinical trial registration number:**

Registration number P2117, study number NL77191.028.21.

**Supplementary Information:**

The online version contains supplementary material available at https://doi.org/10.1007/s40618-026-02943-y.

## Introduction

Androgens, or anabolic androgenic steroids (AAS), are a group of compounds that encompass testosterone and its derivatives. Androgen abuse is widespread, with global lifetime prevalence estimates ranging from approximately 1 – 6% among men [1, 2]. Androgen abuse is harmful as it may cause a broad spectrum of adverse health outcomes, including hepatic toxicity, cardiovascular disease [3], endocrine dysfunction [4] and psychiatric morbidity [5, 6].

In addition to these well-established risks, androgen exposure may adversely affect sleep. The relationship between androgen exposure and sleep-disordered breathing, particularly obstructive sleep apnea, has been debated for several decades [7–10]. Most available evidence originates from therapeutic contexts involving testosterone replacement therapy rather than from real-world androgen abuse. A comprehensive review of this literature concluded that testosterone therapy may exert adverse effects on sleep-disordered breathing, although findings are inconsistent and conflicting across studies [11]. Sleep-disordered breathing may lead to symptoms such as excessive daytime sleepiness, headaches and snoring, and poses significant health risks including metabolic disturbances and cardiovascular morbidity [12]. Given that cardiovascular disease is a major adverse outcome associated with androgen abuse, androgen-related exacerbation of sleep-disordered breathing may represent a potential mechanistic pathway linking androgen exposure to cardiovascular risk.

In a real-world setting, the doses of androgens abused are generally five to twenty times higher than those used in therapeutic or experimental contexts [13, 14]. As a result, the extent to which the existing literature on testosterone therapy can be extrapolated to populations engaging in androgen abuse is uncertain. Data specifically addressing sleep outcomes in individuals abusing supraphysiological doses of androgens is scarce. To our knowledge, the only study directly assessing sleep-disordered breathing in this population is a small cross-sectional investigation by Venancio et al. which did not demonstrate differences between androgen users and trained or sedentary control groups [15].

Given these limited findings, the effects of supraphysiological androgen abuse on breathing-related sleep disorders, sleep architecture and sleep quality remain unclear. The present study is the first prospective investigation in male recreational androgen abusers to evaluate these characteristics of sleep during an androgen cycle.

## Methods

This study was embedded within a larger prospective trial evaluating the efficacy of a harm-reduction intervention in androgen abusers [16]. The study protocol was approved by the local Medical Ethical Committee (MEC registration P2117, study number NL77191.028.21) in June 2021, after which inclusion commenced. All participants provided written informed consent prior to participation. The harm reduction intervention did not include components specifically targeting sleep or sleep-disordered breathing [17].

### Participants

Men aged 18 years or older who intended to initiate a self-administered androgen cycle within two weeks of providing informed consent were eligible for inclusion. Prior to enrolment, participants had to indicate a planned cycle of at least six weeks with a cumulative weekly androgen dose of ≥ 250 mg. Exclusion criteria included the diagnosis of a clinically significant somatic or psychiatric disorder within six months prior to inclusion, initiation or significant modification of treatment for a chronic condition, psychiatric ward or hospital admission for any reason other than physical trauma. Men who had abused androgens within three months prior to inclusion were excluded, with the exception of individuals using a testosterone maintenance dosage of ≤ 250 mg/week, reflecting commonly reported maintenance dosages that are intended to prevent hypogonadal symptoms rather than to induce supraphysiological exposure. Individuals with a prior diagnosis of sleep apnea syndrome or other sleep disorders were excluded. Participants were excluded from the present analysis if sleep data were unavailable for either baseline (T0) or during the androgen cycle (T1).

For the subgroup analysis, participants were classified according to their patterns of androgen exposure across the three assessment time points. Participants who conducted an androgen cycle and were androgen-free at both T0 and T2 were categorized as intermittent androgen abusers. Participants who reported abuse of a testosterone maintenance dose < 250 mg/week at T0 and/or T2 were categorized as continuous androgen abusers (so-called ‘blast and cruise’ protocol). Luteinizing hormone (LH) concentrations were measured to corroborate self-reported androgen abuse status, as supraphysiological androgen exposure induces negative feedback–mediated suppression of LH secretion, thereby providing an objective marker of recent androgen abuse.

### Sample size and power calculation

Among men aged 30 to 60 years, the prevalence of an apnea–hypopnea index (AHI) ≥ 5 has been estimated at 24% [12, 18]. The study was powered to detect a doubling of the prevalence of AHI ≥ 5 during androgen abuse. Assuming a type I error rate of 5% (α = 0.05) and a type II error rate of 20% (β = 0.20; power = 0.80), the required total sample size was calculated to be 27 participants.

### Study procedures

Participants attended the Elisabeth-TweeSteden Hospital (Tilburg, the Netherlands) on three occasions during the study period: prior to initiation of the androgen cycle (T0), during the cycle at approximately week 8 (T1) and approximately three months after cycle cessation (T2). At each visit, among others, detailed information regarding performance- and image enhancing drug abuse was obtained. Participants were instructed on the use of an ambulatory sleep diagnostic device (WatchPAT One®; Itamar, Caesarea, Israel) and were asked to perform the assessment on a night representative of their usual circadian rhythm. Participants were instructed to abstain from alcohol on the day of measurement.

The WatchPAT device records peripheral arterial tone (PAT) signals, oxygen saturation, heart rate, actigraphy, snoring, and body position, from which automated indices including the PAT respiratory distress index (pRDI), PAT apnea–hypopnea index (pAHI), PAT oxygen desaturation index (pODI), and sleep stage were generated. To improve alignment with polysomnography, all data were subsequently reviewed and manually scored by an experienced scorer [19]. The scorer was blinded to the timing of assessment.

At each visit, participants completed two validated sleep questionnaires to assess subjective sleep experiences. Daytime sleepiness was evaluated using the Epworth Sleepiness Scale (ESS). Insomnia symptoms were assessed using the Insomnia Severity Index (ISI), which captures difficulties with sleep initiation and maintenance, early awakening, associated distress, and functional impairment. In addition, a non-validated general sleep quality questionnaire evaluated participants’ self-reported sleep-related symptoms, including perceived sleep quality, daytime fatigue, snoring and symptoms suggestive of restless legs.

### Outcome measures

The primary outcome was longitudinal change in pAHI across time points (T0, T1, T2). Secondary outcomes included the changes in pRDI, pODI, pAHIc, total sleep time, sleep latency, REM-latency, sleep stage distribution, sleep efficiency, number of awakenings, mean heart rate, mean oxygen saturation, snoring threshold and total scores on the ISI and ESS.

### Statistical analysis

Simple descriptive statistics were used to summarize quantitative data. Longitudinal changes in sleep parameters were analysed using linear mixed-effects models with time point (T0, T1, T2) included as a fixed effect and participant as a random intercept, to account for within-subject correlation across repeated measurements. Separate models were fitted for each sleep outcome. In addition, subgroup analyses were conducted to account for differences in androgen abuse patterns, comparing participants engaging in intermittent cycles with those following a continuous regimen. Analyses were performed using Statistical Package for the Social Sciences (SPSS) version 26.0 (IBM Corp.) software.

## Results

### Clinical characteristics

**Fig. 1:**
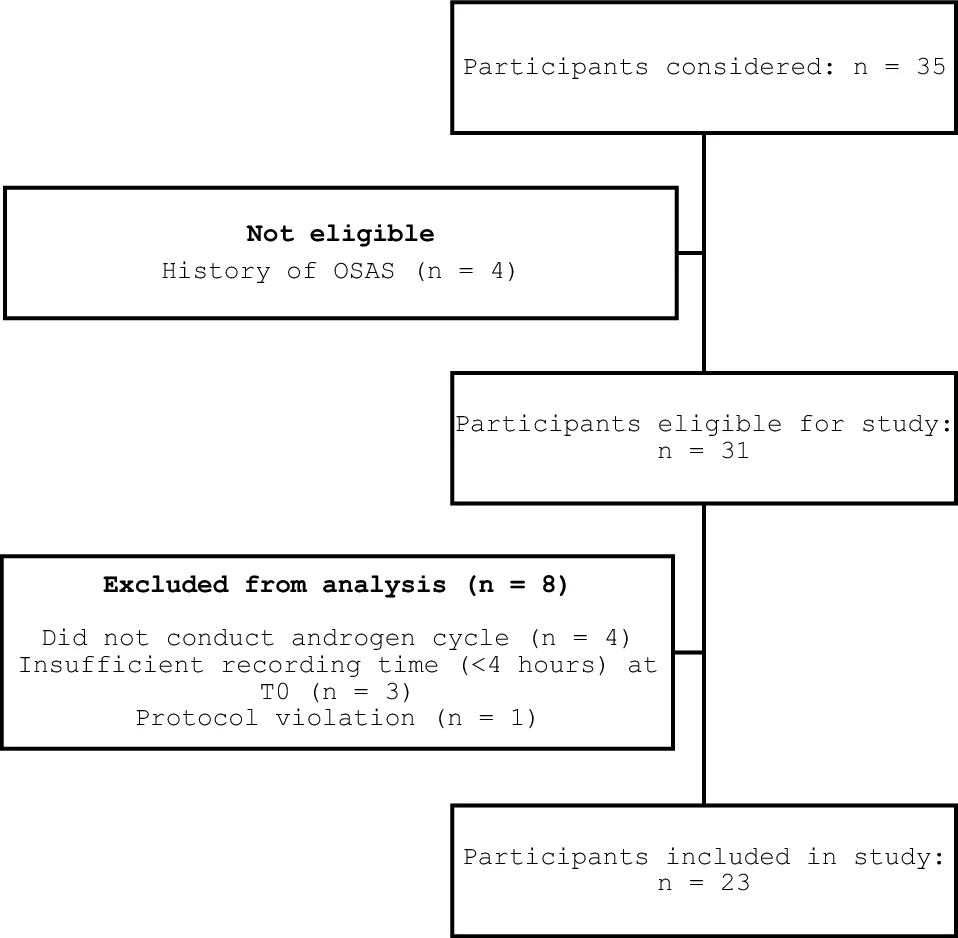
Flow chart of participant inclusion

The first 26 eligible participants from the parent harm reduction trial were enrolled in the present study, after which five additional consecutively eligible participants were included to increase the sample size. Of the 31 enrolled participants, 23 were included in the final analyses after attrition (Fig. 1).

**Fig. 2:**
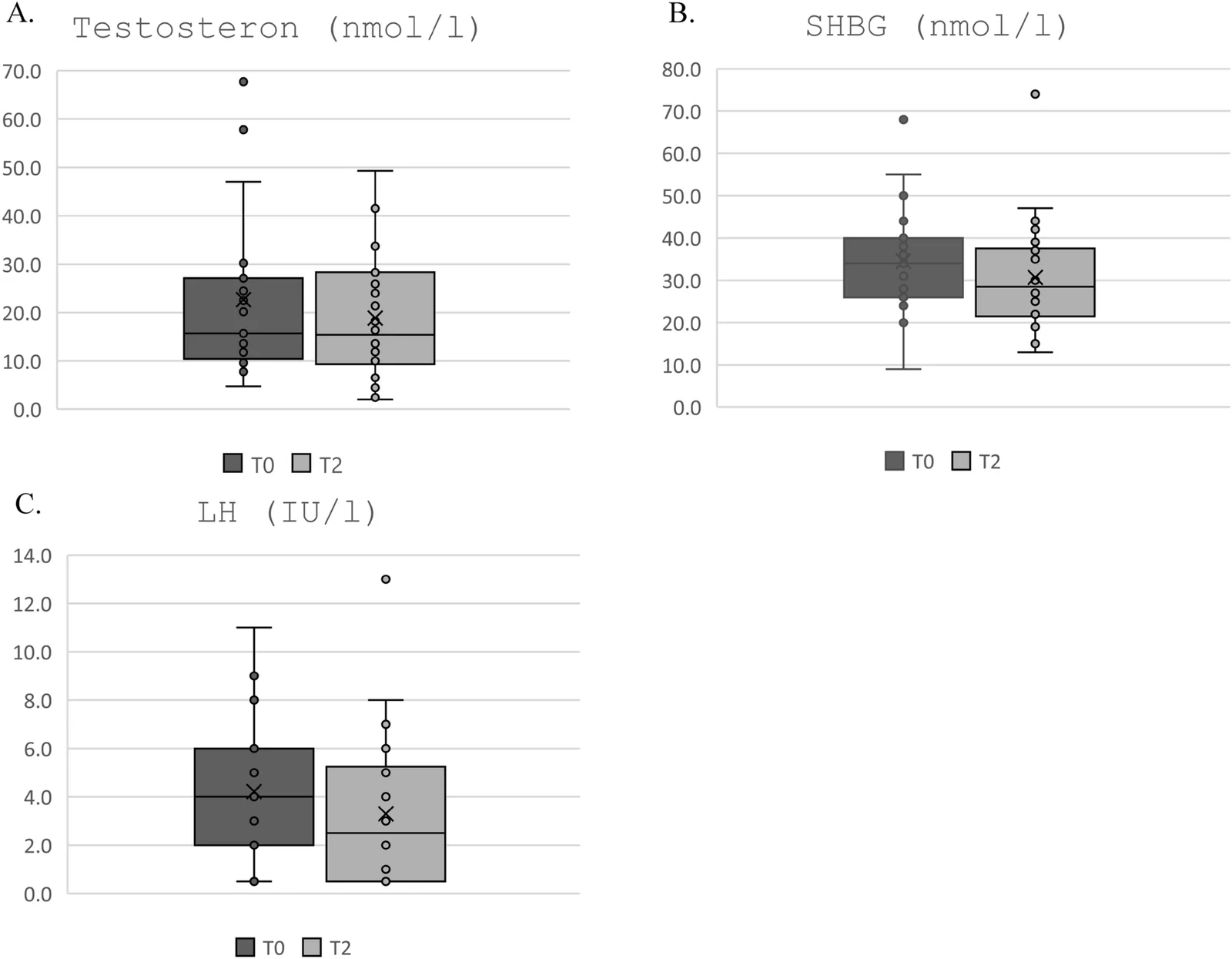
Box-and-whisker plots showing changes in (**A**) total testosterone, (**B**) sex hormone- binding globulin, and (**C**) luteinizing hormone (LH) before (T0) and after (T2) the androgen cycle. LH values below the limit of detection (1 IU/L) were assigned a value of 0.5 IU/L

Baseline characteristics and androgen cycle parameters of all participants are presented in Table 1. When stratified by subgroup, 19 participants engaging in intermittent cycles reported a median weekly dose of 600 mg/week (IQR 350–800) over a median duration of 12 weeks (IQR 10 – 12). The median cumulative cycle dose was 7200 mg (4000 – 8300). Four participants were continuous androgen abusers, who used a median weekly dose of 902 mg/week (IQR 833–1015) over a median duration of 12 weeks (IQR 9 – 12). The median cumulative cycle dose was 10,125 mg (8210 – 12,175). Their testosterone maintenance dosage was on average 175 mg/week (144 – 213). LH concentrations remained above the threshold of suppression (defined as the lower limit of detection of the LH assay of 1 IU/L) in all participants reporting abstinence at T0 or T2, whereas all participants reporting ongoing androgen abuse at T0 and/or T2 had suppressed LH levels, consistent with exogenous androgen exposure. Results of endocrine measurements are presented in Fig. 2.

**Table 1:** Baseline characteristics

General	n (%), median (IQR)
Male	23 (100%)
Age (years)	30.3 (28.6 to 37.6)
Height (cm)	183 (180 – 187)
Weight (kg)	89.1 (80.1 – 98.2)
Body Mass Index (kg/m^2^)	25.8 (24.0 to 28.9)
Smoking (< 3 months) (n, %)	4 (17%)
Alcohol (< 3 months) (n, %)	4 (17%)
**Historic androgen abuse**	
First-time users	3 (13%)
Cumulative duration of androgen abuse among participants with historic abuse (months)	8.4 (3.9 – 29.3)
Continuous androgen abusers	4 (17%)
**Current androgen abuse**	
Weekly dose (mg)	678 (380 – 875)
Cycle duration (weeks)	12 (10 – 12)
Cumulative cycle dose (mg)	7800 (4000 – 10,115)
**Baseline blood markers**	
LH (IU/l)	4 (2.5 – 5.5)
SHBG (nmol/L)	34 (27 – 40)
Testosterone (nmol/L)	15.7 (10.6 – 25.8)

IQR = interquartile range

### Changes in sleep-disordered breathing indices, sleep architecture and questionnaires

Pairwise comparisons demonstrated modest increases in indices of sleep-disordered breathing over time (Table 2). During active androgen abuse (T1), the increase in pAHI (+ 2.1 events/hour; 95% Confidence Interval [95% CI] -0.2 to 4.5; p = 0.075) did not reach statistical significance. A similar non-significant change was observed for pODI (+ 2.4 events/hour; 95% CI -0.1 to 4.9; p = 0.059). pRDI increased significantly compared with baseline (+ 3.0 events/hour; 95% CI 0.2 to 5.7; p = 0.035). At post-cycle follow-up (T2), pAHI was significantly higher than baseline (+ 2.7 events/hour; 95% CI 0.3 to 5.2; p = 0.029). Similarly, pRDI and pODI were also significantly increased with changes of + 4.4 events/hour (95% CI 1.6 to 7.3; p = 0.003) and + 3.0 events/hour for pODI (95% CI 0.5 to 5.6; p = 0.021), respectively.

**Table 2:** Differences in sleep parameters

All participants	T0 (n = 23)	T1 (n = 23)		T2 (n = 21)	
	Mean (95%CI)	Mean (95%CI)	Difference T0 (95%CI)	Mean (95%CI)	Difference T0 (95%CI)
pAHI (events/hour)	3.7 (1.5 – 5.9)	5.9 (3.7 – 8.1)	2.1 (-0.2 – 4.5)	6.4 (4.2 – 8.7)	2.7 (0.3 – 5.2)*
pRDI (events/hour)	7.9 (5.1 – 10.7)	10.8 (8.1 – 13.6)	3.0 (0.2 – 5.7)*	12.3 (9.5 – 15.2)	4.4 (1.6 – 7.3)**
3% pODI (events/hour)	3.6 (1.4 – 5.8)	6.0 (3.8 – 8.2)	2.4 (-0.1 – 4.9)	6.6 (4.4 – 8.9)	3.0 (0.5 – 5.6)*
pAHIc (events/hour)	0.2 (-0.0 – 0.4)	0.4 (0.1 – 0.6)	0.2 (-0.1 – 0.5)	0.3 (0.1 – 0.6)	0.2 (-0.1 – 0.5)
Average oxygen saturation (%)	95.4 (95.1 – 95.8)	95.3 (94.9 – 95.7)	-0.1 (-0.5 – 0.2)	95.1 (94.7 – 95.5)	-0.3 (-0.7 – 0.1)
Total sleep time (min)	366 (339 – 393)	360 (333 – 387)	-6 (-38 – 27)	358 (330 – 386)	-8 (-41 – 26)
Sleep latency (min)	16 (13 – 19)	20 (17 – 22)	4 (-0 – 8)	18 (15 – 21)	2 (-2 – 6)
REM-latency (min)	83 (55 – 111)	117 (89 – 145)	34 (-6 – 74)	96 (66 – 126)	13 (-28 – 55)
Light sleep stage (%)	56 (52 – 60)	57 (53 – 60)	1 (-4 – 5)	54 (50 – 58)	-2 (-6 – 3)
Deep sleep stage (%)	23 (21 – 25)	20 (18 – 23)	-3 (-5 – -0)*	23 (21 – 25)	0 (-3 – 2)
REM sleep stage (%)	21 (18 – 24)	23 (20 – 26)	2 (-1 – 6)	23 (20 – 26)	2 (-2 – 5)
Sleep efficiency (%)	85 (82 – 88)	83 (80 – 86)	-2 (-4 – 1)	85 (82 – 87)	0 (-3—3)
Number of wakes (n)	9 (7 – 11)	10 (8—11)	1 (-1 – 3)	9 (7 – 11)	0 (-2 – 2)
Average heart rate (bpm)	57 (53 – 61)	61 (57—65)	4 (1 – 7)*	57 (53 – 61)	1 (-3 – 4)
Insomnia Severity Index	6.8 (4.6 – 9.1)	7.0 (4.8 – 9.3)	0.2 (-2.2 – 2.6)	6.9 (4.5 – 9.2)	0.0 (-2.5 – 2.5)
Epworth Sleepiness Scale	6.5 (4.5 – 8.5)	7.0 (5.0 – 9.1)	0.5 (-1.0 – 2.1)	6.1 (4.1 – 8.2)	-0.4 (-2.0 – 1.2)
Total body weight	89.3 (84.9 – 93.8)	94.3 (89.9 – 98.8)	5.0 (-1.1 – 11.2)	91.1 (86.6 – 95.6)	1.7 (-4.4 – 7.9)
BMI	26.4 (25.1 – 27.7)	27.9 (26.7 – 29.0)	1.4 (-0.2 – 3.1)	26.8 (25.6 – 27.9)	0.4 (-1.3 – 2.1)

The presented means are estimated marginal means derived by linear mixed-effects models. T0 = at the start of the cycle, T1 = approximately week 8 of androgen cycle, T2 = approximately 3 months after the end of the androgen cycle. ^*^ p < .05, ^**^p < .01, ^***^p < .001. CI = confidence interval

Regarding sleep architecture, no statistically significant differences were observed in the proportions of light sleep or REM sleep during androgen abuse compared with baseline. The proportion of deep sleep was modestly lower during androgen abuse (-3%; 95% CI -5 to -0; p = 0.022). Moreover, average heart rate increased during the androgen cycle (+ 4 beats/minute; 95% CI 1 to 7; p = 0.017). No statistically significant changes were observed across pAHIc, total sleep time, sleep latency, REM latency, sleep efficiency, number of awakenings, mean oxygen saturation or total scores on ISI or ESS questionnaires. General sleep questionnaire outcomes are summarized in Supplementary Table 1.

To explore potential dose–response associations, exploratory analyses were conducted using linear regression models with within-subject change scores (ΔT1–T0 and ΔT2–T0) in pAHI, pODI and pRDI as dependent variables and weekly androgen dose as a continuous predictor. Weekly androgen dose was not associated with changes in pAHI during the androgen cycle (β =  − 0.258 events/hour per 100 mg; 95% CI − 0.9 to 0.4; p = 0.409) or at post-cycle follow-up (β =  − 0.191 events/hour per 100 mg; 95% CI − 1.0 to 0.7; p = 0.641). Similarly, no evidence of dose-dependent associations was observed for pRDI or pODI.

### Subgroup analysis

When stratified by androgen abuse pattern, intermittent androgen abusers (n = 19) demonstrated modest changes in several sleep parameters (Table 3). During active androgen abuse (T1), no significant differences were observed in pAHI or other sleep-disordered breathing indices. Sleep efficiency decreased slightly (− 3%; 95% CI -5 to -1; p = 0.008) and average heart rate increased (+ 5 beats/min; 95% CI 2 to 8; p < 0.001). At post-cycle follow-up (T2), pAHI did not differ significantly from baseline, whereas pRDI was significantly increased (+ 3.6 events/hour; 95% CI 0.3 to 6.9; p = 0.034).

**Table 3:** Differences in sleep architecture and sleep-disordered breathing measures between baseline (T0) and follow-up time points (T1 and T2) in the intermittent androgen abuse subgroup

Intermittent androgen abuse	T0 (n = 19)	T1 (n = 19)		T2 (n = 17)	
	Mean (95%CI)	Mean (95%CI)	Difference T0 (95%CI)	Mean (95%CI)	Difference T0 (95%CI)
pAHI (events/hour)	4.0 (1.5 – 6.5)	5.8 (3.3 – 8.4)	1.9 (-0.8 – 4.6)	6.2 (3.6 – 8.8)	2.2 (-0.6 – 5.1)
pRDI (events/hour)	8.3 (5.0 – 11.7)	11.1 (7.8 – 14.5)	2.8 (-0.4 – 6.0)	11.9 (8.5 – 15.3)	3.6 (0.3 – 6.9)*
3% pODI (events/hour)	3.9 (1.3 – 6.4)	6.0 (3.5 – 8.5)	2.2 (-0.8 – 5.1)	6.5 (3.8 – 9.1)	2.6 (-0.4 – 5.)
pAHIc (events/hour)	0.2 (-0.0 – 0.5)	0.4 (0.1 – 0.7)	0.2 (-0.2 – 0.5)	0.4 (0.1 – 0.7)	0.2 (-0.2 – 0.5)
Average oxygen saturation (%)	95.5 (95.1 – 95.8)	95.3 (94.9 – 95.6)	-0.2 (-0.6 – 0.1)	95.1 (94.7 – 95.5)	-0.4 (-0.7 – -0.0)*
Total sleep time (min)	375 (346 – 403)	360 (331 – 388)	-15 (-51 – 21)	354 (334 – 394)	-10 (-47 – 27)
Sleep latency (min)	17 (13 – 20)	19 (16 – 22)	2 (-2 – 7)	19 (15 – 22)	2 (-3 – 6)
REM-latency (min)	80 (49 – 112)	122 (90 – 153)	41 (-2 – 85)	82 (48 – 116)	2 (-44 – 48)
Light sleep stage (%)	55 (51 – 50)	57 (52 – 61)	2 (-3 – 6)	54 (49 – 58)	-1 (-6 – 4)
Deep sleep stage (%)	23 (20 – 26)	20 (18 – 23)	-3 (-6 – -0)*	23 (20 – 25)	0 (-3 – 2)
REM sleep stage (%)	22 (19 – 25)	23 (20 – 26)	1 (-2 – 4)	24 (21 – 26)	2 (-2 – 5)
Sleep efficiency (%)	87 (84 – 89)	83 (81 – 86)	-3 (-5 – -1)**	85 (83 – 88)	-1 (-4—1)
Number of wakes (n)	8 (6 – 10)	10 (8—12)	2 (-0 – 4)	9 (7 – 11)	1 (-1 – 4)
Average heart rate (beats per min)	54 (50 – 58)	59 (55—63)	5 (2 – 8)***	54 (50 – 58)	1 (-3 – 4)
Insomnia Severity Index	6.7 (3.8 – 9.7)	7.2 (4.2 – 10.2)	0.5 (-3.5 – 4.5)	6.5 (4.2 – 8.8)	-0.3 (-3.8 – 3.3)
Epworth Sleepiness Scale	6.3 (4.2 – 8.3)	6.6 (4.1 – 9.1)	0.3 (-3.4 – 2.8)	6.1 (3.5 – 8.7)	-0.2 (-3.4 – 3.0)
Total body weight	87.8 (83.1 – 92.5)	93.4 (88.4 – 98.5)	5.7 (-1.0 – 12.3)	89.9 (84.8 – 94.9)	2.1 (-4.5 – 8.8)
BMI	26.0 (24.6 – 27.3)	27.6 (26.4 – 28.8)	1.6 (-0.1 – 3.4)	26.2 (25.1 – 27.4)	0.2 (-1.5 – 1.9)

CI = confidence interval

Among participants following a continuous androgen abuse regimen (n = 4), no significant changes in pAHI or other sleep-disordered breathing parameters were observed during the cycle (T1), although sleep latency increased (+ 10 min; 95% CI 0 to 23; p = 0.048) (Table 4). At post-cycle follow-up (T2), pAHI remained unchanged. pRDI increased significantly compared with baseline (+ 8.2 events/hour; 95% CI 2.7 to 13.8; p = 0.011), while the number of nocturnal awakenings decreased (− 5 awakenings; 95% CI -10 to -1; p = 0.026).

**Table 4:** Differences in sleep architecture and sleep-disordered breathing measures between baseline (T0) and follow-up time points (T1 and T2) in the continuous androgen abuse subgroup

Continuous androgen abuse	T0 (n = 4)	T1 (n = 4)		T2 (n = 4)	
	Mean (95%CI)	Mean (95%CI)	Difference T0 (95%CI)	Mean (95%CI)	Difference T0 (95%CI)
pAHI (events/hour)	2.5 (-3.7 – 8.6)	6.0 (-0.2 – 12.1)	3.5 (-2.4 – 9.4)	7.4 (1.3 – 13.5)	5.0 (-0.9 – 10.8)
pRDI (events/hour)	5.7 (1.4 – 10.0)	9.5 (5.2 – 13.8)	3.8 (-1.8 – 9.3)	13.9 (9.6 – 18.2)	8.2 (2.7 – 13.8)*
3% pODI (events/hour)	2.4 (-3.7 – 8.4)	5.9 (-0.2 – 12.0)	3.6 (-2.1 – 9.3)	7.5 (1.4 – 13.5)	5.1 (-0.6 – 10.8)
pAHIc (events/hour)	0.0 (-0.2 – 0.2)	0.2 (0.0 – 0.3)	0.2 (-0.0 – 0.4)	0.1 (-0.1 – 0.2)	0.1 (-0.2 – 0.3)
Average oxygen saturation (%)	95.3 (93.5 – 97.0)	95.5 (93.8 – 97.2)	0.3 (-1.6 – 2.1)	95.3 (93.5 – 97.0)	0.0 (-1.8 – 1.8)
Total sleep time (min)	325 (233 – 416)	363 (271 – 455)	38 (-62 – 139)	330 (238 – 422)	5 (-95 – 106)
Sleep latency (min)	12 (5 – 19)	23 (15 – 30)	10 (0 – 23)*	15 (8 – 22)	3 (-8 – 13)
REM-latency (min)	94 (18 – 170)	93 (17 – 169)	-1 (-102 – 99)	151 (75 – 227)	57 (-44 – 157)
Light sleep stage (%)	60 (52 – 69)	56 (48 – 65)	-4 (-16 – 8)	55 (46 – 63)	-6 (-18 – 6)
Deep sleep stage (%)	24 (19 – 29)	21 (16 – 26)	-3 (-10 – 4)	26 (20 – 31)	2 (-5 – 8)
REM sleep stage (%)	16 (6 – 25)	23 (13 – 33)	7 (-7 – 21)	20 (10 – 30)	4 (-10 – 18)
Sleep efficiency (%)	77 (64 – 89)	81 (68 – 93)	4 (-8 – 17)	82 (70 – 94)	5 (-7—18)
Number of wakes (n)	14 (10 – 17)	10 (6—13)	-4 (-8– 0)	9 (5 – 12)	-5 (-10 – -1)*
Average heart rate (beats per min)	71 (65 – 77)	68 (61—73)	-4 (-12 – 5)	72 (66 – 77)	0 (-8 – 9)
Insomnia Severity Index	7.3 (-0.6 – 15.1)	6.3 (0.1 – 12.4)	-1.0 (-8.8 – 6.7)	7.3 (0.1 – 14.4)	0.0 (-8.2 – 8.2)
Epworth Sleepiness Scale	7.8 (-2.4 – 17.9)	9.3 (3.1 – 15.4)	1.5 (-8.1 – 11.1)	6.0 (2.1 – 9.9)	-1.8 (-11.4 – 7.9)
Total body weight	96.9 (79.1 – 114.6)	98.6 (82.7 – 114.4)	1.8 (-16.6 – 20.1)	96.5 (81.3 – 111.8)	-0.3 (-18.4 – 17.8)
BMI	28.6 (23.1 – 34.0)	29.1 (24.2 – 33.9)	0.5 (-5.2 – 6.2)	28.5 (23.2 – 33.7)	-0.1 (-6.1 – 5.0)

CI = confidence interval

## Discussion

Androgen abuse was associated with statistically significant but modest changes in sleep-disordered breathing parameters, while sleep architecture was largely preserved. During active androgen abuse, the primary outcome pAHI showed a small increase compared with baseline that did not reach statistical significance (p = 0.075). This finding should be interpreted with caution, as the sample size was smaller than planned and may have limited statistical power to detect modest effects. A similar non-significant increase was observed for pODI (p = 0.059), while pRDI increased significantly during androgen abuse. At post-cycle follow-up, statistically significant increases were observed for pAHI. At this time point, pRDI, and pODI were also significantly increased.

Notably, the most consistent changes in sleep-disordered breathing were observed after androgen exposure. This suggests that the effects on sleep-disordered breathing persist for several months beyond androgen abuse. A similar temporal pattern has been reported in prior work describing poorer subjective sleep quality during periods of androgen abstinence, although objective sleep measurements were not performed [20].

Several mechanisms may underlie the changes observed. Supraphysiological androgen exposure is known to induce alterations in body composition, including increases in fluid retention and lean muscle mass [21]. When occurring in the neck and upper airway, such changes may increase upper airway collapsibility during sleep and thereby promote obstructive respiratory events. Importantly, these changes may resolve only gradually after cessation of abuse, which could account for the apparent worsening of sleep-disordered breathing indices at post-cycle follow-up (T2).

The timing of the assessments may also be relevant. The during-cycle measurement (T1) was conducted at approximately week 8 of the androgen cycle, and may not have captured the full impact of sustained supraphysiological androgen exposure. It is plausible that more pronounced effects on sleep-disordered breathing would have emerged later in the cycle, as androgen concentrations remain elevated and changes in body composition develop.

Beyond structural mechanisms, androgens may also influence ventilatory control through effects on respiratory drive and chemosensitivity, potentially altering the balance between ventilatory stability and upper airway patency [22]. The absence of changes in pAHIc suggests that the observed effects are unlikely to be driven by central apneas, supporting a predominantly obstructive mechanism.

Hypogonadism has also been associated with obstructive sleep apnea syndrome in previous studies [23, 24]. This may be relevant in the context of androgen withdrawal, as intermittent androgen abusers typically experience a transient period of low testosterone levels following cycle cessation. This could potentially have contributed to the observed increase in breathing-related parameters during the post-cycle period. Notably, endocrine parameters had largely recovered by T2, whereas breathing-related parameters remained elevated, suggesting a possible lag-time effect or, alternatively, that transient hypogonadism may not explain the observed findings.

The findings from our study show partial overlap with the only previous study, to our knowledge, that specifically investigated sleep-disordered breathing in recreational androgen abusers. In this cross-sectional study, Venancio et al. (2008) compared recreational androgen abusers with control participants using polysomnography and reported no differences in apnea–hypopnea index between groups, which aligns with the absence of a clear effect on pAHI during the present study. However, the authors reported reduced sleep efficiency and an increased number of awakenings after sleep onset. In the present study, these observations were not observed in the overall cohort, although a modest reduction in sleep efficiency was detected among intermittent abusers. In addition, we observed a small reduction in deep sleep during androgen abuse, a finding that was not reported in the cross-sectional study [15].

This study has several strengths. It is the first prospective investigation to evaluate the effects of androgen abuse on sleep-disordered breathing parameters. By enrolling men conducting self-administered androgen cycles at median doses approximately eightfold higher than standard therapeutic levels, the study is representative of real-world androgen abuse, thereby enhancing the generalizability of results. In addition, the use of ambulatory sleep monitoring with the WatchPAT device enables convenient and user-friendly data collection in a home environment while maintaining acceptable diagnostic accuracy compared to laboratory polysomnography [25].

From a clinical perspective, it is important to note that our findings cannot be extrapolated to patients with hypogonadism initiating testosterone replacement therapy, particularly those with pre-existing obstructive sleep apnea. Guidelines recommend caution in hypogonadal males due to the potential for worsening of pre-existing sleep-disordered breathing [24, 26]. While the present study suggests only modest effects of androgen doses several times higher than those used during testosterone replacement therapy, it does not provide direct evidence regarding the safety of testosterone therapy in patients with hypogonadism and obstructive sleep apnea, given that our cohort consisted of predominantly eugonadal individuals and excluded those with a prior diagnosis of sleep-disordered breathing.

Several limitations warrant consideration. First, the modest sample size (n = 23) limited statistical power to detect small changes and increased the risk of type II errors. This is particularly relevant when interpreting the non-significant findings for the primary outcome pAHI, which showed an increase during active androgen abuse that did not reach statistical significance. Nevertheless, even when statistically significant, the observed effect sizes were small and unlikely to be clinically meaningful. In addition, the subgroup of continuous androgen abusers was small (n = 4), which precludes drawing meaningful conclusions from the analyses of this subgroup. Second, androgens were obtained from the unregulated market, introducing uncertainty regarding the exact compound composition and dosage [27, 28]. Although standardized prescribing could have solved this limitation, this would be medically and ethically inappropriate and would not be representative of real-world androgen abuse. Third, while the ambulatory sleep device has been validated against polysomnography, it may be less sensitive to subtle changes in sleep stage transitions and microarousals compared with full laboratory-based polysomnography [25]. Fourth, unmeasured confounding factors such as neck size, training intensity, dietary patterns, psychological stress, and concurrent substance use may have influenced sleep outcomes. Fifth, the exclusion of participants with a known diagnosis of obstructive sleep apnea may have introduced selection bias. Finally, participants in the present cohort had relatively modest body mass index values compared to those typically observed in high-level bodybuilders or strength athletes. It is therefore possible that individuals with a higher body mass index may be more susceptible to androgen-related changes in sleep-disordered breathing, limiting the generalizability of the findings to such populations.

## Conclusion

In this prospective cohort of male recreational androgen abusers, androgen abuse was associated with adverse changes in indices of sleep-disordered breathing in men, while sleep architecture remained largely preserved, and perceived sleep quality did not change. Although statistically significant increases in pAHI, pRDI and pODI were observed, the absolute magnitude of these changes was small, rendering their clinical relevance uncertain. These findings suggest that androgen abuse may subtly influence sleep-disordered breathing parameters, but are unlikely to translate into overt or clinically meaningful sleep apnea in most androgen abusers.

## Supplementary Information

Below is the link to the electronic supplementary material.

Supplementary file 1

## Data Availability

The data that support the findings of this study are available from the corresponding author, TV, upon reasonable request.
