# Supplementary material for: Effect of androgen abuse on sleep apnea in men: a prospective cohort study
Source: J Endocrinol Invest. 2026 Jul 20;49(10):2785–93. doi: 10.1007/s40618-026-02943-y (PMC13627260; doi:10.1007/s40618-026-02943-y)
Supplement: Supplementary file 1 [file 40618_2026_2943_MOESM1_ESM.docx]

***Supplementary Table 1:*** *Results of the general sleep questionnaire.*

| **All participants** | **Response category** | **T0 (n=23)**  **n (%)** | **T1 (n=23)**  **n (%)** | **T2 (n=21)**  **n (%)** |
| --- | --- | --- | --- | --- |
| Thoughts racing at night | None \| None \| \| \| | 9 (39%) | 9 (39%) | 14 (67%) |
|  | \| Moderate \| \| \| | 10 (44%) | 13 (57%) | 7 (33%) |
|  | \| Severe \| \| \| | 4 (17%) | 1 (4%) | 0 (0%) |
| Vivid dreams / nightmares | None \| None \| \| \| | 16 (70%) | 17 (74%) | 17 (81%) |
|  | \| Moderate \| \| \| | 6 (26%) | 5 (22%) | 3 (14%) |
|  | \| Severe \| \| \| | 1 (4%) | 1 (4%) | 1 (5%) |
| Morning headache \| None \| \| \| | None \| None \| \| \| | 21 (91%) | 20 (87%) | 19 (91%) |
|  | \| Moderate \| \| \| | 2 (9%) | 3 (13%) | 2 (10%) |
|  | \| Severe | 0 (0%) | 0 (0%) | 0 (0%) |
| Feeling insufficiently rested upon awakening | Nearly every day | 4 (17%) | 3 (13%) | 3 (14%) |
|  | 3–4 times per week | 3 (13%) | 3 (13%) | 2 (10%) |
|  | 1–2 times per week | 9 (39%) | 7 (30%) | 7 (33%) |
|  | 1–2 times per month | 5 (22%) | 7 (30%) | 6 (29%) |
|  | Never / almost never | 2 (9%) | 3 (13%) | 3 (14%) |
| Daytime fatigue / reduced alertness | Nearly every day | 3 (13%) | 1 (4%) | 2 (10%) |
|  | 3–4 times per week | 5 (22%) | 3 (13%) | 6 (29%) |
|  | 1–2 times per week | 7 (30%) | 8 (35%) | 3 (14%) |
|  | 1–2 times per month | 4 (17%) | 7 (30%) | 8 (38%) |
|  | Never / almost never | 4 (17%) | 4 (17%) | 2 (10%) |
| Daytime sleepiness | Not | 5 (22%) | 5 (22%) | 11 (52%) |
|  | Mild | 16 (70%) | 16 (70%) | 9 (43%) |
|  | Moderate | 1 (4%) | 2 (9%) | 1 (5%) |
|  | Severe | 1 (4%) | 0 (0%) | 0 (0%) |
| Concentration difficulties | Not | 11 (48%) | 10 (44%) | 11 (52%) |
|  | Mild | 8 (35%) | 12 (52%) | 7 (33%) |
|  | Moderate | 3 (13%) | 1 (4%) | 3 (14%) |
|  | Severe | 1 (4%) | 0 (0%) | 0 (0%) |
| Impaired daytime functioning | Normal | 17 (74%) | 17 (74%) | 16 (76%) |
|  | Mildly impaired | 4 (17%) | 5 (22%) | 5 (24%) |
|  | Moderately impaired | 2 (9%) | 0 (0%) | 0 (0%) |
|  | Severely impaired | 0 (0%) | 0 (0%) | 0 (0%) |
|  | Missing data | 0 (0%) | 1 (4%) | 0 (0%) |
| Snoring | Yes | 6 (26%) | 6 (26%) | 6 (29%) |
|  | No | 11 (48%) | 10 (44%) | 10 (48%) |
|  | Unknown | 5 (22%) | 7 (30%) | 5 (24%) |
|  | Missing data | 1 (4%) | 0 (0%) | 0 (0%) |
| Awakening with choking / dyspnea | Yes | 22 (96%) | 22 (96%) | 19 (91%) |
|  | No | 1 (4%) | 1 (4%) | 2 (10%) |
| Perceived sleep quality | Sufficient | 12 (52%) | 9 (39%) | 10 (48%) |
|  | Slightly insufficient | 7 (30%) | 9 (39%) | 9 (43%) |
|  | Moderately insufficient | 2 (9%) | 4 (17%) | 2 (10%) |
|  | Severely insufficient | 2 (9%) | 1 (4%) | 0 (0%) |
| Unpleasant leg sensations or an urge to move legs | Yes | 22 (96%) | 23 (100%) | 20 (95%) |
|  | No | 1 (4%) | 0 (0%) | 1 (5%) |
